# Comprehensive Planning for Classification and Disposal of Solid Waste at the Industrial Parks regarding Health and Environmental Impacts

**DOI:** 10.1155/2014/230163

**Published:** 2014-02-13

**Authors:** Hassan Hashemi, Hamidreza Pourzamani, Bahareh Rahmani Samani

**Affiliations:** ^1^Department of Environmental Health Engineering, Faculty of Health, Shahrekord University of Medical Sciences, Shahrekord 88157-13471, Iran; ^2^Environment Research Center, Isfahan University of Medical Sciences, Isfahan 81676-36954, Iran; ^3^Islamic Azad University Sciences and Research Branch of Ahwaz, Ahwaz 61555-163, Iran

## Abstract

The aim of this study is the comprehensive planning for integrated management of solid waste at the industrial parks. The share of each industrial group including food, metal, chemical, non-metallic minerals, textile, electrical and electronical, and cellulose industries were 48.2, 14.9, 6.7, 22, 0.9, 0.6, and 6.5 percent, respectively. The results showed that nearly half of total industrial waste produced from the range of biological materials are biodegradable and discharging them without observing environmental regulations leads to short-term pollution and nuisance in the acceptor environment. Also some parts of case study waste were recyclable which is considerable from viewpoint of economical and environmental pollution. Long-term impacts will appear due to improper site selection of disposal from the spatial standpoint. In this way, an approach for site selection using several socioeconomic, physical, and environmental criteria based on multicriteria decision making model (MCDM) is introduced. Health risks and environment pollution such as soil and surface water may be done. It is essential to revise the studied industries layout, particularly those units which produce special waste which should be more cautious. Also stricter enforcement is required as an effective step in reducing the harmful impacts of it.

## 1. Introduction

Industrial growth, technological advancements, and higher living standards in today's society contribute to the ever increasing generation of solid wastes.

Industrial park is one of the most important manufacturing bases and an indicator of development in each country. However, each manufacturing process usually generates some amount of nonconsumable waste causing adverse effects on environment [1].

Landfill sites, particularly in developing countries, pose significant health and environmental problems. Attention to the appropriate landfill site selection according to environmental inventory is important. Geographical information system (GIS) technologies are effectively used in the process of site selection, which is a spatial problem [2].

Despite the consensus of all countries to achieve the goal of zero waste in industries, the accomplishment of this goal is predicted to be difficult and generation of various wastes arising from manufacturing processes is currently inevitable [3].

A focus on sources of solid waste with respect to management is justified by the fact that waste characteristics and composition differ according to source [4].

The appearance of such problems caused some countries to move their waste to developing countries in order to be saved from the probable risks of the hazardous waste [5]. Uncontrolled expansion of industries and ignoring environmental principles in industrial development and overuse have caused much environmental problems [6]. A number of countries have ratified state and national legislation for managing and controlling hazardous waste whose main purpose is to minimize the potential risks of industrial hazardous waste for humans and the environment [7]. In Iran, despite the excess growth of industries, urbanization, and industrial centralization around major cities particularly Tehran, no fundamental effort has been yet made in the field of industrial waste disposal, and subsequently, there are few rules for controlling and disposal of such materials [8]. Evidence shows that solid, semisolid, and liquid wastes of the factories are disposed regardless of health issues and effluent of these industries enters the absorbing wells or spread in surrounding land. Solid waste is piled up around the factories for a period, and then a part of the waste is incinerated outdoor and some part is transferred out of the factories along with household waste by municipal general service. Large scale land use transportation from agricultural to industrial has made Tehran a city for a large center for industries centralization and their peripheral activities. Because of the importance of industrial hazardous waste minimization in prevention of pollutant emissions in the environment, it is emphasized by current laws in many countries. Moreover, establishment and development of minimization programs have been adopted more than ever through legislation of laws and more restrictions in controlling hazardous waste and prevention of leaving waste in the environment [9]. The application of waste minimization techniques does not necessarily mean the use of complicated technologies or investments on expensive machines. Many methods require a simple change in the process of production or in transferring the materials. Generally, hazardous waste minimization methods can be used for any production process and the mutual factor in all techniques is the reduction of production costs [10].

In this study, basic and technical information required for the waste management were collected and analyzed due to the increasing growth of industries and the need for developing efficient methods in proportion to the conditions of the studied region. In fact, the main objective of this study is to examine the quantity of industrial solid waste regarding the production line of CB province, Iran. Then the criteria for site selection were listed.

## 2. Method and Materials

The population of this study included all operational industries in 19 industrial zones of CB province, Iran, which were studied for 8 months from march to october, 2009. Data were collected partly through questionnaires and partly through field investigation and literature review on the subject on matter, which was carried out using various books and articles. Field investigation was done using questionnaires in studied industrial zones. For each industrial unit a questionnaire was completed accurately. Industrial zones of the province were identified in order to specify the production resources. In this regard, detailed information on the industrial zones was taken from the Iran Small Industries and Industrial Parks Organization [4], in CB province. There were 19 approved industrial zones with the total area of 3356.10 hectares. Metal industries with 95 units and electricity and electronics industries with 10 units had maximum and minimum numbers of units in the province, respectively [6]. The first stage of the study including separation and classification of the industrial units was done based on their activities and locations according to the available statistics in the province. The questionnaire contained type of industrial group, the number of personnel, the kind of product and raw materials, the amount and type of generated waste, waste physical state, frequency of generation, method of storage and maintenance, recycling methods, final disposal, and the responsible organizations for waste collection in order to identify industrial waste in CB province. Various questionnaires presented by different organizations such as Recycle Organization, Environmental Protection Organization, and Ministry of Industries were analyzed and finally a questionnaire containing two forms was prepared. All the data were analyzed using SPSS, Excel, and GIS.

## 3. Results and Discussion

According to the investigations, 19 industrial parks with the total area of 2356.10 hectares were under the coverage of Industrial Parks Organization. Among these 19 industrial parks, 14 have been operated. shahrekord, Lordegan, Saman, Faradnbeh, Junghan, Farokhshahr, Ben, and Dastgerd industrial parks had 154, 22, 16, 1, 2, 3, 4, and 3 units, respectively. Therefore, shahrekord with 154 units (or 49.8%) and Faradnbeh with 1 unit (or 0.3 percent) had the most and the least units, respectively. A number of 309 questionnaires were completed by the industrial units. Generally, these industries performed various activities, so the amount of industrial waste was influenced by the kind of activities. In this regard, these industries were divided according to the classification of Ministry of Industries. Food, metal, chemical, nonmetallic minerals, cellulose, textile, and electrical and electronics industries comprised 21, 30.4, 22, 12.6, 7.1, 3.8, and 2.9 percent of the evaluated industries in this study. The workers of these 14 industrial parks were 6033 people. Based on Table 1, the total industrial waste was 1246 tons per month, and semihousehold waste per capita (for food consumption of the workers) was 350 gram per day.  Table 2 shows that the amount of wastes generated in CB province was approximately 32 ton per month.

According to Table 3, maximum and minimum wastes are generated in food and electrical industries, respectively.

The total food waste generated in studied industries was an average of 65516 kg per month which depended on the number of workers. Therefore, food waste per capita was 350 gram per day. Herbal waste was 384 tons a month of which 99.4% and 0.6% were generated in food and chemical industries, respectively. Herbal waste of food industries encompassed wheat, straw, bran, fruit, vegetable, and dried fruit waste. Herbal waste of chemical industries was related to yarn and fabric dyeing industries. In these industries, herbal colors were used for dyeing and subsequently, some herbal wastes were generated. Flour and doughy waste was generated in industries like macaroni manufacturing, noodle making, and industrial bread. The amount of flour and doughy waste was 22.3 tons per month in the province which was totally generated in food industries. Sweets, chocolate, and candy waste was 6.5 tons per month which was totally generated and recycled in food industries. Meat and bone waste encompassed chicken and fish packaging waste and sausage production waste which was totally generated in food industries. According to investigations, wood waste was generated in metal, chemical, cellulose, and electricity and electronics industries in a form of wooden pallets which entered the factories along with purchase of tools like the engines of various machines and variety of metal ingots. The amount of wood waste was 17 tons per month, of which the maximum amount of 55.2 percent was related to metal industries. Plastic waste comprising mainly packaging in industries was 28.3 tons per month. Chemical industries generate the maximum amount of plastic waste of 53.8 percent. The amounts of plastic waste generated in food, metal, nonmetallic minerals, cellulose, textiles, and electrical and electronics industries were 40.8, 1.9, 0.8, 0.3, 0.3, and 2.2 percent, respectively. These types of waste were recycled in chemical industries; however, no action has been yet done for recycling these wastes by other industries. A few food industries were willing to sell plastic waste.

The amount of glass waste in the entire study was 8.2 tons per month of which 91.8, 6, and 2.2 percent were generated in nonmetallic minerals, cellulose, and food industries, respectively. The amount of paper and cardboard waste was 84.5 tons per month of which 83.1 percent was generated in cellulose industries and 12.2 percent was generated in food and other industries. Metal waste was divided into two categories of ferrous and nonferrous metals. Ferrous metals waste included iron chips, iron scraps, and snipping which was 160 tons per month of which 94.8, 6, and 0.3 percent were generated in metal and cellulose industries, respectively. Nonferrous metal waste included aluminum, copper, and cast iron of which the maximum amount was related to electricity and electronics industries for the use of aluminum and copper in wire and cable industry. The amount of nonferrous metal waste in studied samples was 5.7 tons per month of which 52.2, 31.4, 8.7, 1.2, and 3.5 percent were generated in electricity and electronics, metal, cellulose, nonmetal, and food industries, respectively. Gunny waste consisting of sugar and flour sacks generated 4003 kg per month of which 81.9, 15.6, and 2.5 percent were generated from food, chemical, and metal industries. It should be noted that 100 percent of these wastes were reused and exploited in the same industries. Trash waste included concrete, mosaics, ceramics, and stone body waste which were totally generated in nonmetal industries. The amount of generated trash waste was 250 tons per month. The amount of generated PVC waste in the studied zones was 2.5 tons per month of which chemical, and electricity and electronics industries comprised 2300 kg (91.6 percent) and 70 kg (2.8 percent), respectively. The amount of generated PET waste was 18 tons (93.7 percent) per month of which 200 kg (1.1 percent) and 935 kg (5.2 percent) were generated in metal and food industries, respectively. Other industries did not generate PET waste. The amount of polystyrene waste was 5.8 tons per month which was totally generated in chemical industries. The amount of polypropylene waste was 6.2 tons per month of which 96.8 percent and 3.2 percent were generated in chemical and textile industries, respectively. Soil waste consisting of wheat soil and bentonite clay was used for vinegar treatment in industrial vinegar-making and stabilization of edible oils. Furthermore, burnt sand waste (CO_2_ silica sand CO_2_ waste) is contained in soil waste. The amount of soil waste generated in the province was approximately 130 tons per month of which 96.6% and 3.4% were generated in food and metal industries, respectively. As noted above, burnt sand waste was classified in this category and was generated in cast iron foundry industry.

The amount of yarn and fabric waste in all studied zones was about 7.4 tons a month. These wastes included yarn and fabrics, wool, fiber, and polymer of which 93.9 percent and 6.1 percent were generated in textile and chemical industries. The amount of chemical and special waste in all studied zones was about 21 tons per month of which 3, 41, 36.9, 16.6, 2, and 4 percent were generated in metal, chemical, nonmetal, cellulose, textile, and electricity and electronics industries. The initial chemical waste produced from industries which produced detergents and disinfectants comprised 1 percent of the raw chemical materials. These wastes consisted of sulfuric acid, diethanolamine, coconut fatty acid, betaine, essence, resin, sulfate, salt, and so forth. The amount of these wastes was 1775 kg per month which comprised 8.1 percent of the total chemical waste. The amount of color waste was 581 kg per month which comprised 2.6 percent of the total special waste. It should be noted that this type of waste was found in metal, chemical, cellulose, and textile industries. The amount of adhesives waste was 172 kg per month which was related to chemical and cellulose industries and was most generated in card board industry. Adhesives waste comprised 0.8 percent of total waste of this category. Sludge waste includes the sludge from wastewater of industries. Acidic sludge was 18330 kg per month. A study on the industries of industrial parks in other region of Iran showed that 250 kg of waste containing degradable materials and scrap iron was transferred out of the city and incinerated outdoor by municipal service. Overestablishment of industries and conversion of agricultural land to industrial land in Tehran, Iran, have changed the city to a large center for industries centralization and their peripheral activities. In this study, the maximum amount of generated sludge waste was 8 tons per month which was related to nonmetal industries that was generated in a mosaic-building industrial unit in Boroujen industrial zone. The tar waste generated in the province was 220 kg per month which was related to the waterproof membrane industries. The tar waste comprised 1% of total special waste. The zinc oxide waste was 35 kg per month which comprised 0.1 percent of total special waste in the studied industrial parks. Moreover, zinc clay waste was generated by a zinc ingot production cooperative in ShahreKord. It was 600 kg per month which comprised 2.7 percent of total chemical and special waste.

An ideal waste disposal site is that one which is located fairly close to the source of the waste, has easy transportation access, is not located in a low-lying area or floodplain, and is underlain by geologically stable, strong, and competent rock material [11].

The site selection depends on several factors like land use, environmental, hydrology, socioeconomic, and so forth [12].

According to the literature, the following criteria can be applied for site selection [13]:

- site must be close to at least a street with a buffer of 30 m;
- site must not be too far from a transfer station;
- site must be 3 km from residential areas, with the exception of areas with barriers;
- there should be a minimum distance of 100 m between site and roads;
- site must be on a suitable soil;
- site should be constructed in areas which do not have an important economic or ecological value.

**Figure 1:**
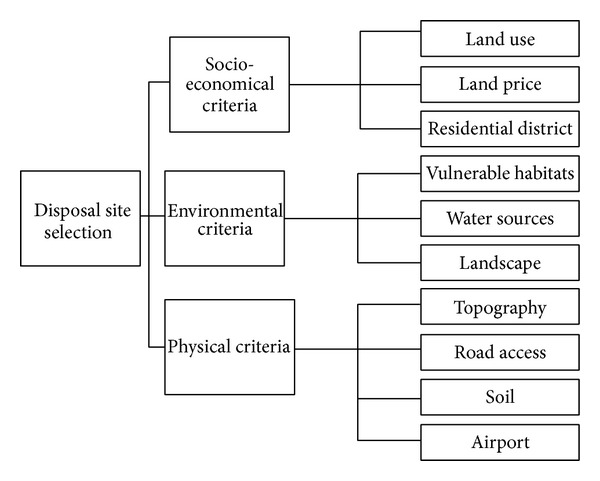
Hierarchal structure for disposal site selection problem.

Such kinds of criteria could be combined in a hierarchal structure shown in Figure 1.

The combination of GIS and multicriteria decision models (MCDM) can be useful in this regard. Such approach provides flexible methods for exploring relationships among geographic data and assisting experts from diverse fields in pooling their knowledge to solve complex problems. A list of potential sites which generally satisfy the minimum requirements can be identified for the purpose of effective sanitary landfill-type waste disposal. Among these areas, selection should be made through careful field checks. The integration of GIS in multicriteria decision analysis (MCDA) is a powerful tool in solving disposal site selection problem, because it provides efficient spatial data manipulation and presentation.

## 4. Conclusions

One of the most important issues in industrial waste management is its special waste which distinguishes the industrial waste from the general waste. Therefore, the type of waste should be noticed in hazardous waste management. The waste of this category includes chemical raw materials, color, adhesives, tar, zinc oxide, zinc clay, and sludge [14]. The total generated waste in the studied industrial zones was 1246 tons per month, of which 48.2, 14.9, 6.7, 22, 0.9, 0.6, and 6.5 percent were generated in food, metal, chemical, nonmetallic minerals, textile, electrical and electronics, and cellulose industries, respectively. The results achieved in this study are considered the first step toward proper industrial waste management especially in recycling and disposal of waste [15]. According to the results of this study, Environmental General Department recommended to use rewards and punishments for reduction of hazardous waste. Coordination with financial institutions for giving loans with lower interests in order to take environmental actions or imposing large fines for each kilogram of generated hazardous waste gives instances of reward and punishment. Collection, delivery, and disposal of hazardous waste should be done by private section and the officials of Environmental General Department supervise the performance of the private section. Costs of monitoring water, soil, and weather analyses in hazardous waste disposal site should be adopted from pollutant industries and the environmental report on the landfill should be submitted to Environmental Department every three months. Regarding fragile ecosystem of CB province, Layout and extension of industrial parks in future should be done based on land use planning criteria. Also appropriate management of generated industrial solid wastes is very important. Finally, an approach to the integration of GIS and MCDM was introduced as a powerful tool in solving disposal site selection problem considering several criteria.

## Figures and Tables

**Table 1:** The amount of waste for each industrial zone.

Industrial zone	Weight (kg/per month)	Percentage
Shahrekord	502,159	40.2
Lordegan	15,707	1.2
Saman 1	42,896	3.4
Saman 2	45,260	3.6
Sefid Dasht	172,330	13.8
Boroujen	295,704	23.7
Hafshejan	77,200	6.1
Tishniz	1,120	0.08
Boldaji	6430	0.5
Faradonbeh	1650	0.13
Junghan	1250	0.1
Farokhshahr	77970	6.2
Ben	5520	0.44
Dastgerd	1555	0.1

Total	1246751	100

**Table 2:** The amount and types of solid wastes generated in Chaharmahal and Bakhtiari province.

Type of waste	Amount (kg/per month)	Percentage
Color	581	2.6
Adhesives	172	0.8
Chemical material	1,775	8.1
Sludge	18,330	84.4
Zinc oxide	35	0.1
Zinc clay	600	2.7
Tar	220	1

Total	31,713	100

**Table 3:** The amount of generated waste (kg) and the percentage of generation for each industrial zone.

Industry/waste	Food	Percent	Metal	Percent	Chemical	Percent	Nonmetal	Percent	Cellulose	Percent	Textile	Percent	Electricity	Percent	Total	Percent
Semihousehold	17926	27.4	16590	25.3	16835	25.7	5670	8.7	3310	5.1	3360	5.1	1825	2.8	65,516	100
Herbal	384520	99.4	—	—	2300	0.6	—	—	—	—	—	—	—	—	386,820	100
Flour	22315	100	—	—	—	—	—	—	—	—	—	—	—	—	22,315	100
Sweets	6590	100	—	—	—	—	—	—	—	—	—	—	—	—	6,590	100
Meat	15075	100	—	—	—	—	—	—	—	—	—	—	—	—	15,075	100
Wood	900	5.2	8960	52.2	4955	28.9	—	—	2130	12.4	—	—	210	1.2	17,155	100
Plastics	11557	40.8	540	1.9	15250	53.8	215	0.8	80	0.3	90	0.3	620	2.2	28,352	100
Glass	180	2.2	—	—	—	—	7600	91.8	500	6	—	—	—	—	8,280	100
Card board	10271	12.2	223	0.3	1377	1.6	2170	2.6	70250	83.1	10	Little	215	0.3	8,4516	100
Ferrous metals	1480	0.9	152105	94.8	2290	1.4	1500	0.9	545	0.3	—	—	2485	1.5	160,405	100
Nonferrous metals	200	3.5	1805	31.4	—	—	70	1.2	500	8.7	—	—	3175	55.2	5,750	100
Gunny	3280	81.9	100	2.5	623	15.6	—	—	—	—	—	—	—	—	4,003	100
Trash	—	—	—	—	—	—	249400	100	—	—	—	—	—	—	249,400	100
PVC	—	—	140	5.6	2300	91.6	—	—	—	—	—	—	70	2.8	2,510	100
PET	935	5.2	200	1.1	16870	93.7	—	—	—	—	—	—	—	—	18,005	100
Polystyrene	—	—	—	—	5830	100	—	—	—	—	—	—	—	—	5,830	100
polypropylene	—	—	—	—	6000	96.8	—	—	—	—	200	3.2	—	—	6,200	100
Polluted soil	126336	96.6	4500	3.4	—	—	—	—	—	—	—	—	—	—	130,836	100
Chemical	—	—	649	2.9	8895	40.8	8000	36.8	3614	16.6	550	2.5	100	0.4	21,713	100
Yarn and fabrics	—	—	—	—	460	6.1	—	—	—	—	7020	93.9	—	—	7,480	100

Total waste	601565	48.2	185812	14.9	83985	6.7	274625	22	80929	6.5	11230	0.9	8700	0.7	1246,751	100
